# Salt Mediated Self-Assembly of Poly(ethylene glycol)-Functionalized Gold Nanorods

**DOI:** 10.1038/s41598-019-56730-2

**Published:** 2019-12-30

**Authors:** Hyeong Jin Kim, Wenjie Wang, Wei Bu, Md Mir Hossen, Alejandra Londoño-Calderon, Andrew C. Hillier, Tanya Prozorov, Surya Mallapragada, David Vaknin

**Affiliations:** 1 Ames Laboratory, and Department of Chemical and Biological Engineering, Iowa State University, Ames, Iowa 50011 United States; 2 Division of Materials Sciences and Engineering, Ames Laboratory, Iowa State University, Ames, Iowa 50011 United States; 3 NSF’s ChemMatCARS, University of Chicago, Illinois, 60637 United States; 4 Ames Laboratory, and Department of Physics and Astronomy, Iowa State University, Ames, Iowa 50011 United States

**Keywords:** Nanoscale materials, Structural properties

## Abstract

Although challenging, assembling and orienting non-spherical nanomaterials into two- and three-dimensional (2D and 3D) ordered arrays can facilitate versatile collective properties by virtue of their shape-dependent properties that cannot be realized with their spherical counterparts. Here, we report on the self-assembly of gold nanorods (AuNRs) into 2D films at the vapor/liquid interface facilitated by grafting them with poly(ethylene glycol) (PEG). Using surface sensitive synchrotron grazing incidence small angle X-ray scattering (GISAXS) and specular X-ray reflectivity (XRR), we show that PEG-AuNRs in aqueous suspensions migrate to the vapor/liquid interface in the presence of salt, forming a uniform monolayer with planar-to-surface orientation. Furthermore, the 2D assembled PEG functionalized AuNRs exhibit short range order into rectangular symmetry with side-by-side and tail-to-tail nearest-neighbor packing. The effect of PEG chain length and salt concentration on the 2D assembly are also reported.

## Introduction

Assembling nanomaterials into two-or three- dimensional (2D or 3D) ordered structures can lead to novel collective physical and chemical properties that cannot be realized in the original bulk nanomaterials^1–3^. By virtue of their potential applications in photonics, electronics, plasmonics, sensing, and catalysis^4–7^, self-assembly of nanoparticles (NPs) has attracted a great deal of research effort. Although considerable attention has been devoted to the self-assembly of isotropic NPs such as quantum dots^8–11^ and spherically-shaped NPs^12–14^, relatively less interest has been given to the assembly of anisotropic NPs such as the rod-shaped NPs^15–17^. Anisotropic nanoparticles exhibit unique shape-dependent properties^18^ such as spin-dependent electron transfer^19^, vibrational coherence^20^, intense plasmonic fields^21^, and tandem catalysis^22^. Gold nanorods (AuNRs) in particular possess essential anisotropic plasmonic characteristics, such that, when assembled, exhibit tunable surface plasmon resonances with local electromagnetic fields that are two to five times more intense than those achieved with spherical AuNPs^23,24^. In addition, these emerging properties can be further controlled by geometric parameters such as the aspect ratio (radius-to-length), size, interparticle distances, and orientation^23,25^.

Although possessing attractive properties, self-assembly of AuNR into organized superstructures remains challenging. For example, the traditional solvent-evaporation method leads to uncontrollable AuNR arrays with random orientations^26^. Thus, other avenues for self-assembly of AuNRs are needed^7^. Several reliable approaches to achieving 2D or 3D ordered structures have recently been demonstrated, including the ligand capping of AuNR^27^, the use of DNA origami as a template^28^, and surfactant-assisted techniques^29^. In addition, AuNRs grafted with poly(ethylene glycol) (PEG) have been assembled into highly packed two dimensional (2D) arrays at the water/vapor interface by spreading and manipulating them as Langmuir monolayers and subsequently transferring them to quartz or silicon wafers by the Langmuir-Blodgett (LB) technique^30^. Another approach took advantage of grafting NRs and nanowires with short chain hydrophobic alkyl chains, and spread and manipulate them at the air-water interface achieving a high degree of orientational order^31–34^.

Here, we report on a readily controlled self-assembly approach grafting of AuNRs with PEG and inducing 2D crystallization by manipulating salt concentrations in the PEG-AuNR suspensions. As has been shown in previous studies with AuNPs, taking advantage of the so-called aqueous biphase systems strategy^35–37^, PEG-functionalized spherical AuNPs have been assembled into 2D and 3D superstructures by controlling ionic strength in aqueous suspensions^38–40^. We thus, examine this approach to assemble AuNRs, and apply surface sensitive synchrotron X-ray techniques including specular X-ray reflectivity (XRR) and grazing incidence small angle X-ray scattering (GISAXS) to characterize the assembly of AuNRs at the vapor/liquid interface. We propose that the method presented here can be potentially further extended to assemble a variety of complex nanostructures such as nanotriangles, nanocubes and other nanostructure shapes^41–43^.

## Results and Discussion

### X-ray reflectivity(XRR)

**Figure 1:**
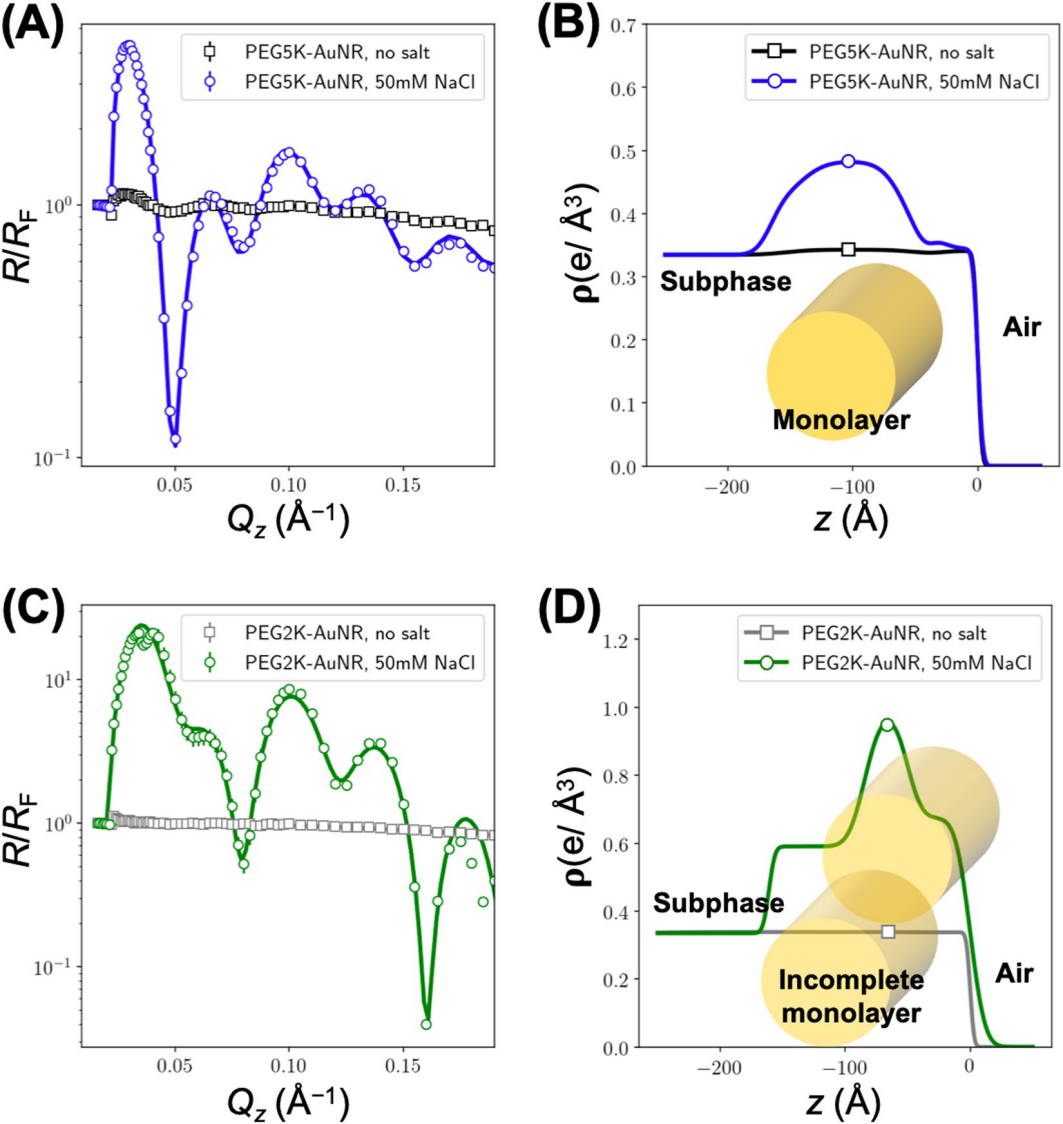
Normalized XRR data for (**A**) PEG5K-AuNRs and (**C**) PEG2K-AuNRs with and without salt as indicated. Solid lines of $R/{R}_{{\rm{F}}}$ in (**A**,**C**) are obtained from one of the best fit electron density (ED) profiles shown in (**B**) PEG5K-AuNRs and (**D**) PEG2K-AuNRs.

Figure 1(A,C) show normalized XRR data, $R/{R}_{{\rm{F}}}$ (where $R$ is the measured reflectivity and ${R}_{{\rm{F}}}$ is the calculated Fresnel reflectivity of an ideally flat vacuum/liquid interface), for PEG5K-AuNRs and PEG2K-AuNRs, with and without 50 mM NaCl. Without the addition of salt, we notice a very weak signal that indicates spontaneous accumulation of minute amounts of PEG-AuNRs at the interface. As expected, this signal is slightly stronger for the longer grafted PEG. The significant change in the $R/{R}_{{\rm{F}}}$ data after the addition of NaCl to the suspension of PEG-AuNRs, is evidence of film formation of PEG-AuNRs at the aqueous surface driven by the salt. We note that the first maximum in $R/{R}_{{\rm{F}}}$ for PEG2K-AuNRs is much higher than that for PEG5K-AuNRs for the same salt concentration (i.e., 50 mM NaCl). This provides evidence that PEG2K-AuNRs accumulate at higher surface density at the vapor/suspension interface than PEG5K-AuNRs.

**Figure 2:**
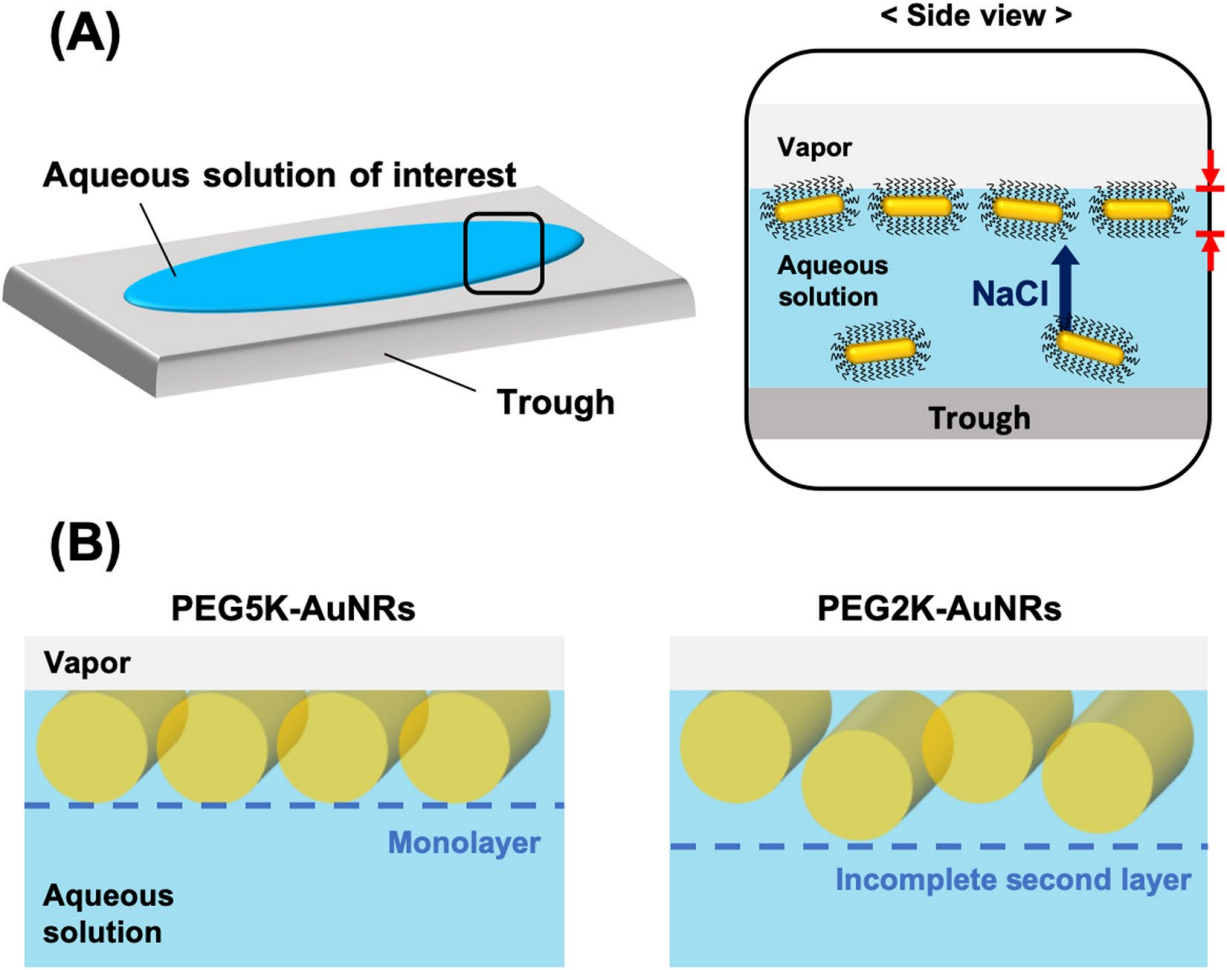
(**A**) Schematic illustration of 2D assembly of PEG-AuNRs at the vapor/liquid interface induced by NaCl, and (**B**) another side view of 2D assembly of PEG-AuNRs in different direction. In (**B**), PEG attached to AuNRs is omitted for clarity.

For quantitative analysis of $R/{R}_{{\rm{F}}}$, we refine an ED profile model that generates a best fit to the $R/{R}_{{\rm{F}}}$ data by using Parratt’s recursive method to calculate the reflectivity^44–46^. Figure 1(B,D) show ED profiles that best fit the corresponding $R/{R}_{{\rm{F}}}$ measured curves. Similar XRR results and ED profiles for PEG5K-AuNRs in 2 M NaCl are shown in Fig. S1(A–D) in the SI. The enhanced ED region is dominated by the high ED of Au (i.e., AuNRs) and is confined to a thickness that is close to the diameter of the AuNRs, indicating the formation of highly uniform single AuNR film with a characteristic thickness that is consistent with the AuNRs lying with their rotational axis parallel to the surface. Figure 2 depicts an illustration of a side view of the monolayer at the suspension/vapor interface that forms after the addition of salt. Since the ED of the grafted PEG-chain region is almost the same as that of the aqueous-subphase, it is impossible to identify the PEG in the profile. While for PEG5K-AuNRs we find that the ED stratum is consistent with the AuNR diameter (see Table 1), for the PEG2K-AuNRs, we find a slightly thicker stratum (~16 nm). We also note that the ED of the second layer is significantly lower than the one at the vapor interface. This suggests the formation of a second incomplete layer of AuNRs that are closely packed with a motif of hexagonal packing of rods as depicted in Fig. 1(D).

**Table 1:** Size distribution of AuNR determined by S/TEM and SAXS. The nominal values are provided by the manufacturer.

(nm)	Nominal	STE/M	SAXS
Diameter	12.5 ± 1.4	12.3 ± 1.4	11.8 ± 0.4
Length	50.8 ± 5.0	49.7 ± 5.3	50.0 ± 9.0

To determine the extent of accumulation of AuNRs at the interface, the excess electron surface density $({\rho }_{2D})$ and average surface coverage are estimated (see more detail in the SI, Fig. S2). Higher ${\rho }_{2D}$ and surface coverage of PEG2K-AuNRs compared to that of PEG5K-AuNRs show that the aqueous surface is more populated with PEG2K-AuNRs.

We note that increasing salt concentration from 50 to 2000 mM in the PEG5K-AuNRs suspension changes only slightly the XRR and the GISAXS, as shown in the Fig. S1(A–D). The estimated increase in AuNRs at the interface is only 12% to 13% surface coverage, and increased salt concentration does not lead to 3D precipitates as observed for PEG-AuNPs^38,39^.

### Grazing indidence small angle X-ray scattering (GISAXS)

**Figure 3:**
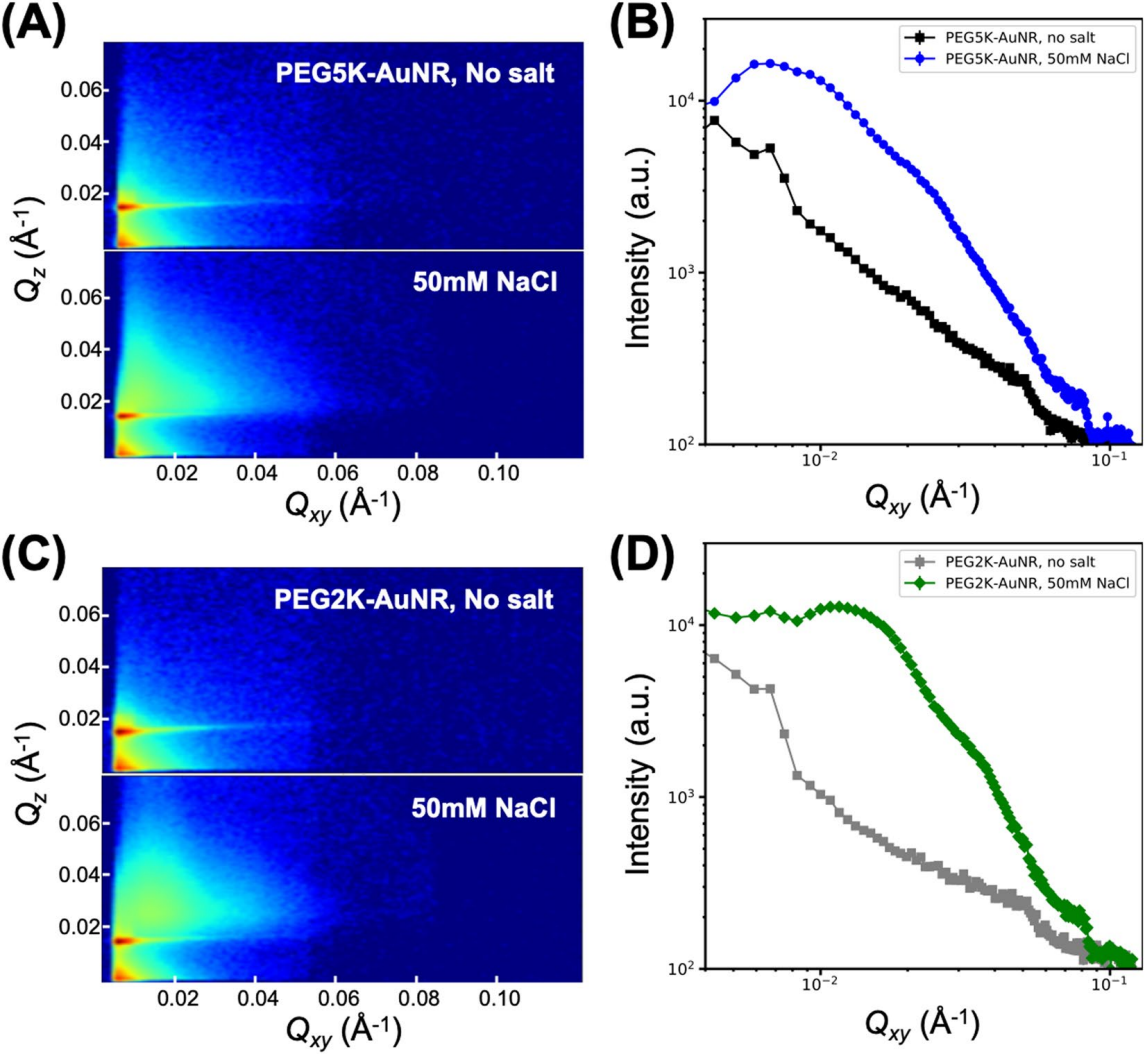
2D GISAXS patterns (${Q}_{xy}$, ${Q}_{z}$) for (**A**) PEG5K-AuNR and (**C**) PEG2K-AuNR suspensions at the vapor/liquid interface with and without salt as indicated. Horizontal ${Q}_{xy}$ linecut profiles (integrated over a ${Q}_{z}$ range from 0.02 to 0.1 Å^−1^) from GISAXS patterns for (**B**) PEG5K-AuNRs and (**D**) PEG2K-AuNRs.

Figure 3(A,C) show 2D GISAXS patterns as a function of ${Q}_{xy}$ and ${Q}_{z}$ for PEG5K-AuNR and PEG2K-AuNR suspensions, respectively. In the absence of NaCl, the GISAXS patterns of PEG-AuNRs display a narrow feature resembling a form-factor of uncorrelated particles, due to the spontaneous accumulation of dispersed minute amount of PEG-AuNRs, consistent with the XRR results discussed above. Upon addition of NaCl, broader GISAXS patterns appear demonstrating that more PEG-AuNRs migrate to the surface and also establish partial in-plane correlations, as discussed below. To quantify the 2D GISAXS images, we plot ${Q}_{xy}$ cuts by integrating the intensity over a ${Q}_{z}$ range from 0.02 to 0.1 Å^−1^ as shown in Fig. 3(B,D). We note that the linecut profiles show a significant increase in intensity following the addition of NaCl. As with the XRR, the higher intensity of PEG2K-AuNRs compared to that of PEG5K-AuNRs indicates that more PEG2K-AuNRs populate the surface.

**Figure 4:**
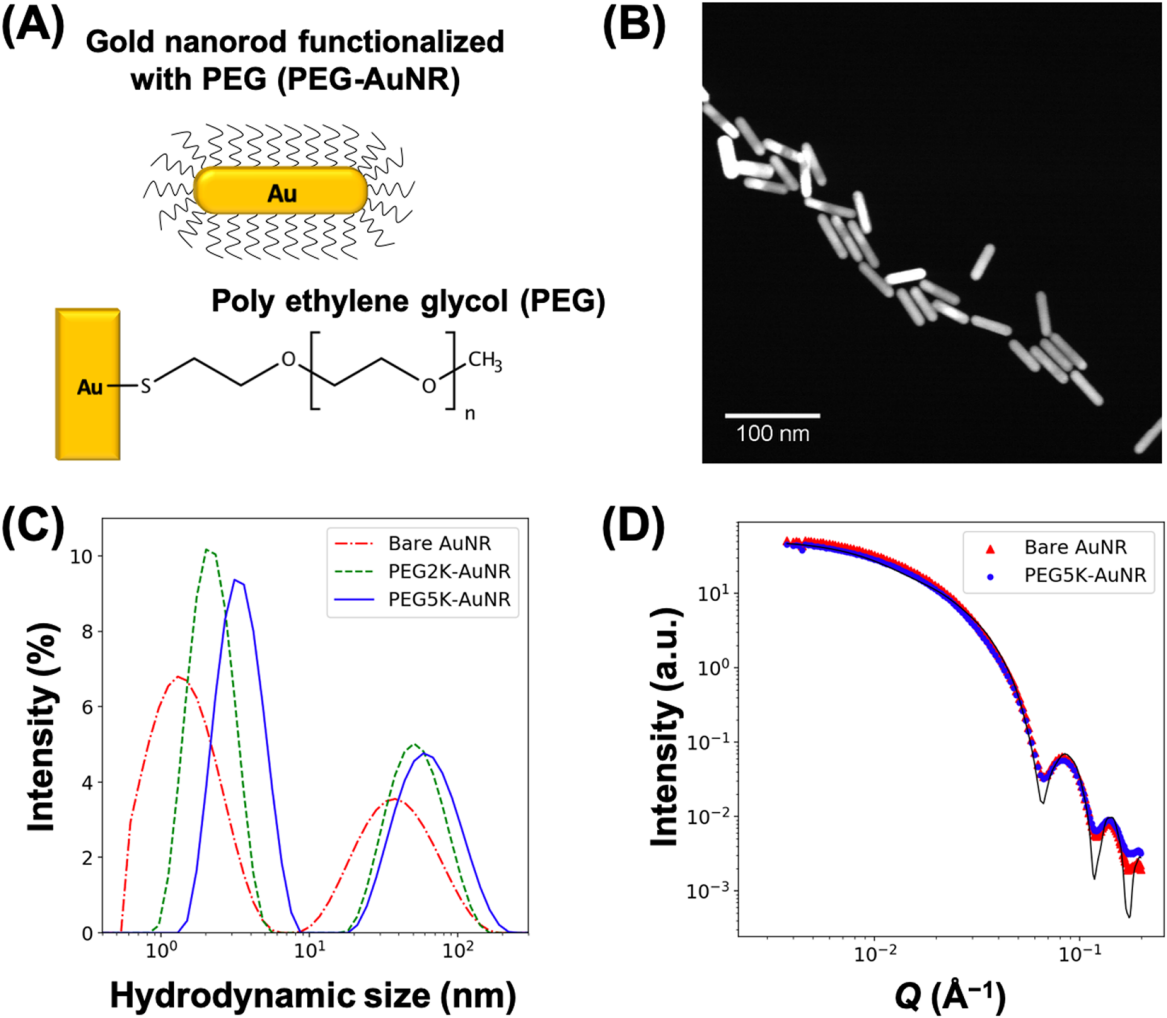
(**A**) Schematic illustration of PEG functionalized AuNRs and (**B**) representative S/TEM image of bare AuNRs. (**C**) DLS data for unfunctionalized (bare) AuNR, PEG2K-AuNR, and PEG5K-AuNR in bulk aqueous solution. (**D**) Small angle X-ray scattering (SAXS) intensity profiles of bare AuNR (red triangles) and PEG5K-AuNR (blue circles). The black solid line is a best fit to SAXS data using the form factor of circular cylinder particles not accounting for the polydispersity of the NRs.

**Figure 5:**
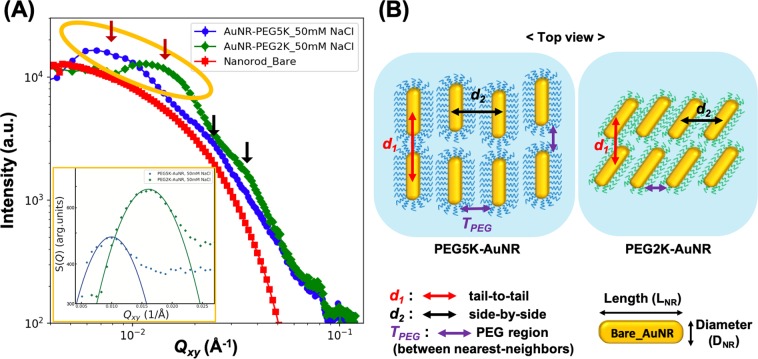
(**A**) Q_*xy*_ linecut profiles of PEG5K-AuNRs (blue circles) and PEG2K-AuNRs (green diamonds) at 50 mM NaCl. The form factor profile for bare AuNRs (red squares) in bulk solution obtained from SAXS experiment is also overlayed. The arrows point to the broad peak positions of PEG5K-AuNRs and PEG2K-AuNRs. The structure factor profile (shown in the inset) of yellow elliptical region is inserted for determination of the first diffraction peak position (*Q*_1_). (**B**) Schematic illustration of 2D assembled PEG5K-AuNRs and PEG2K-AuNRs based on the broad two diffracton peaks shown in (**A**). This is depicted as an ideal long-range 2D crystalline structure.

To further quantify the interfacial 2D assembly of PEG-AuNRs in the presence of NaCl in the suspension, we superimpose the form factor profile of bare AuNRs obtained from SAXS measurements (shown in Fig. 4(D)) for comparison with the ${Q}_{xy}$ cuts, as shown in Fig. 5. Whereas the measured form factor falls smoothly over the plotted range, the linecut profiles for both the PEG-AuNR samples show oscillating features that generally indicate the onset of in-plane ordering. The emergence of two broad interference peaks in the linecut profiles are identified by red and black arrows in Fig. 5. The inset in Fig. 5 shows a portion of the structure factor that is obtained by dividing the measured GISAXS cuts of the PEG-AuNRs by our measured form factor of the AuNRs, yielding a prominent fundamental diffraction peak that we label ${Q}_{1}$ as listed in Table 2. Also listed in Table 2 is the position of the second peak ${Q}_{2}$. Based on the peak position of the broad diffraction peaks, we estimate the *d*-spacings as ${d}_{i}=2\pi /{Q}_{i}$ (where, *i* = 1, 2) as listed in Table 2 for both samples. We suggest that these two *d*-spacings are related to side-by-side and tail-to-tail inter-particle distances of PEG5K-AuNRs reflecting the anisotropy of AuNRs and the planar orientation of the assembled PEG-AuNRs at the interface, as also verified by the XRR results. Assuming the diffraction peaks (${Q}_{1}$ and ${Q}_{2}$) are associated with the tail-to-tail and side-by-side interparticle spacings, respectively, for PEG5K-AuNRs at 50 mM NaCl, the calculated interparticle distances are ${d}_{1}=63.0$ and ${d}_{2}=25.4\,{\rm{nm}}$ implying that PEG5K-AuNR tend to assemble in simple 2D rectangular symmetry. To further examine the validity of our assertion, we evaluate the thickness of the PEG region between nearest-neighbors (${T}_{PEG}$) for PEG5K-AuNRs by subtracting the length ${L}_{NR}$ and diameter ${D}_{NR}$ of a AuNR from ${d}_{1}$ and ${d}_{2}$ respectively, (depicted in Fig. 5(B)). We find that the PEG-region for tail-to-tail ${T}_{PEG}\simeq {d}_{1}-{L}_{NR}=63.4-50.0\simeq 13.4\,{\rm{nm}}$ and for the side-by-side ${T}_{PEG}\simeq {d}_{2}-{D}_{NR}=25-11.8\simeq 13.2\,{\rm{nm}}$. It is interesting to note ${T}_{PEG}$ is approximately the same (~13 nm) in both directions as expected from closely packed PEG-AuNRs. This PEG corona thickness is consistent with that obtained for 2D ordered PEG6K-AuNPs (spherical AuNPs)^38^. This further supports our assertion that ${d}_{1}\ {\rm{and}}\ {d}_{2}$ are simply the lattice constants of the rectangular unit cell that PEG5K-AuNRs tend to assemble into by complying with their shape as depicted in Fig. 5(B).

**Table 2:** Summary of XRR and GISAXS data for PEG5K-AuNRs and PEG2K-AuNRs with 50 mM NaCl.

	*Q*_1_ (Å^−1^)	*Q*_2_ (Å^−1^)	*d*_1_ (nm)	*d*_2_ (nm)	*ρ*_*max*_ (e/Å^3^)	*ρ*_2*D*_ (e/Å^2^)
PEG5K-AuNRs	0.0099 (1)	0.025 (2)	63.4 (3)	25 (2)	0.48 (1)	14.40 (1)
PEG2K-AuNRs	0.0160 (1)	0.036 (1)	39.2 (2)	17.5 (4)	0.98 (6)	56.28 (1)

As expected, the *d*_1_- and *d*_2_-spacings of the assembled PEG2K-AuNRs (39.2 and 17.5 nm, respectively) are smaller than those of the PEG5K-AuNRs, however ${d}_{1}\sim 39\,{\rm{nm}}$ is even smaller than the length of the bare AuNR. We therefore suggest that the grafted PEG2K-AuNRs are tilted in the plane as depicted in Fig. 5(B). Similar estimates of the PEG region of PEG2K-AuNRs to that in PEG5K-AuNRs, yields for side-by-side ${T}_{PEG}\simeq {d}_{2}-{D}_{NR}=$ $17.5-11.8\simeq 5.7$. The ratio of the two PEG regions for PE5K-AuNR ands PEG2K-AuNR ~ $13.3/5.7\simeq 2.3$ roughly resembles the ratio of the molecular weight of the two polymers 2.5.

### Scanning transmission electron microscopy (S/TEM)

**Figure 6:**
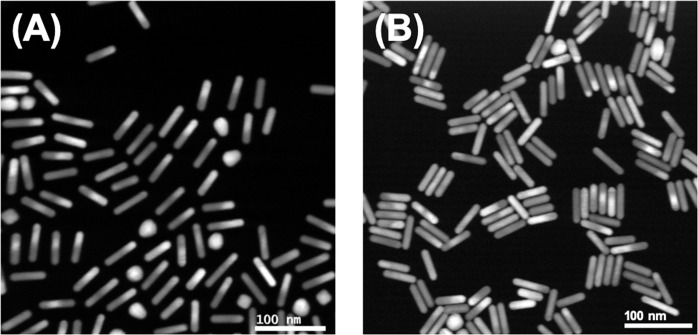
HADDF-S/TEM images of (**A**) PEG5K-AuNRs and (**B**) PEG2K-AuNRs of transferred films from the vapor/liquid interface with 50 mM NaCl to TEM grids.

To corroborate the X-ray diffraction conclusions, we examined electron microscopy images of assembled films of PEG5K-AuNRs and PEG2K-AuNRs that were transferred to solid support from the vapor/liquid interface as described in Methods. Figure 6 shows HAADF-S/TEM images representative of PEG5K-AuNR and PEG2K-AuNR arrangements after adjusting the suspension to 50 mM NaCl. We wish to point out, that while the films lifted from the vapor/suspension interface are likely affected by the transfer process itself, the nanorod arrangement is consistent with our assessment of AuNR arrangement and formation of ordered islands. Further, due to the film being lifted and dried at the surface of the carbon substrate, it is natural to expect breakage in the interfacial films and their long range order upon transfer, yielding the shorter-range film fragments. Importantly, the short-range ordering for two types of AuNRs is distinctively different, and it correlates well with the rest of the experimental results.

It is in fact evident that the dried out assembled clusters in these images (and prevalent across the slide) indicate that the side-by-side and tail-to-tail ordering is to a large extent preserved even after transferring and drying the films. We note that without the addition of salt to PEG5K-AuNRs suspension, the NRs are randomly-dispersed as shown in Fig. S3.

## Conclusions

By grafting AuNRs with PEG, we have demonstrated that PEG-AuNRs can be assembled into 2D films at the vapor/liquid interface induced by salt. Using XRR, GISAXS, and S/TEM we determine the nature of the films that are formed. In the absence of salt, PEG-AuNRs are well dispersed as a suspension and only a minute but detectable amount populates the interface. However, in the presence of salt, PEG-AuNRs accumulate at the interface and form an oriented monolayer such that their axis of rotation is parallel to the liquid/vapor interface. Our XRR shows that the shorter chain PEG2K-AuNRs accumulate at the interface at a higher density than the PEG5K-AuNRs for the same salt concentrations. In addition, the XRR indicates that the PEG2K-AuNR form a corrugated monolayer that may enhance the in-plane packing of AuNRs. Our GISAXS results and S/TEM images suggest that both the PEG5K-AuNRs and PEG2K-AuNRs tend to order in a simple rectangular lattice, with aligned side-by-side and tail-to-tail, conforming to their shape. Whereas both PEG5K-AuNRs tend to align along the major axes of the rectangular lattice, the PEG2K-AuNRs seem to be rotated with respect those axes. Our approach to assemble PEG-AuNRs at the vapor/liquid interface shows the potential to form ordered AuNRs films by adjusting the ionic strength of the suspension. And as demonstrated by the HAADF-S/TEM images, the partially ordered films can, to some extent, preserve their structure after being transferred to solid substrates, that can be further developed for future applications in devices.

## Materials and Methods

### Materials

Citrate-stabilized AuNRs with an aspect ratio of 4.1 (50.8 ± 5 nm length, 12.5 ± 1.4 nm diameter provided by the manufacturer) were purchased from NanoComposix Inc. The shape-purity of the AuNRs is roughly 90% rods and 10% non-rod spheres with the latter typically formed during synthesis of AuNRs^47,48^. Poly(ethylene glycol) (PEG) methyl ether thiol (see Fig. 4(A)) with average molecular weight (${M}_{n}$) of ~2 kDa and ~5 kDa (referred to as PEG2K and PEG5K, respectively) were acquired from CreativePEGWorks Inc. All chemicals were used as received without further purification. AuNRs were functionalized with PEG using a ligand exchange method. Briefly, the PEG methyl ether thiol was dissolved in deionized water (18.2 MΩ/cm^−1^ at 25 °C, Milli-Q water purification system), and mixed with an aqueous suspension of AuNRs (molar ratio of PEG to AuNR ≈ 6000:1) for 3 days on a rotate-shaker. After incubation, PEG-functionalized AuNRs (PEG-AuNRs) were purified by centrifuging (12,000 g force) for 30 min thrice to remove unbound PEG and also to adjust the final concentration of the suspension.

### Methods

The S/TEM images were recorded using FEI Tecnai G2 F20 S/TEM equipped with a Tridiem Gatan image filter operating at 200 kV. We worked in the HAADF S/TEM mode using condenser aperture C1 = 2000 *μ*m, C2 = 70 *μ*m and a camera length of 87 mm, with the data acquisition carried out primarily using a spot size 11. Data analysis was performed with TEM Imaging & Analysis (FEI) software (version 4.12, built 2120). Preparation of EM grids: Continuous carbon support film on Cu 400 mesh grids were purchased from Ted Pella (Redding, CA). All EM grids were placed carbon face up on a glass slide and plasma cleaned with UV ozone Procleaner (Bioforce Nanosciences, Ames, IA, USA) for 15 min before use. The hydrophilized grids were held with the anti-capillary reverse tweezers and ~2 *μ*L of PEG-AuNR solution was placed on top and allowed to stay undisturbed at room temperature for ~1 min, after which time the excess of liquid was gently wicked off using a thin strip of a lens paper. An example of bare AuNRs is shown in Fig. 4(B). Transfer of interfacial PEG-AuNR layer to the grid: A micro-volume of PEG-AuNR solution with NaCl was prepared by placing 2 *μ*l of PEG-AuNR (~1.5 nM) solution in water on a clean hydrophobic corning cover glass with dimensions 1.5 × 22 × 22 mm. To the surface of the droplet, a micro-volume of 4 *μ*l of NaCl, to reach a target NaCl concentration of 50 mM, was added and left to mix for ~1 minute. The water/air surface of the droplet was gently touched with a continuous carbon film grid (carbon face down) for 30 seconds. The hydrophobicity of the substrate and the grid allows collecting material from the surface of the droplet with minimum liquid transfer to the grid, which is then allowed to dry at room temperature before imaging with the STEM. While this approach can be viewed as similar to the Langmuir-Schaefer method for Langmuir films depositions on solid surfaces, the interfacial layer is significantly smaller in size.

Dynamic light scattering (DLS) is used to confirm grafting of the PEG on the AuNRs (Nano ZS90 ZEN3690) by determining the hydrodynamic size distribution of bare AuNR and PEG-functionalized AuNRs. Figure 4(C) shows the hydrodynamic size distribution of AuNR before and after PEG-functionalization. Unlike for spherical AuNPs, the hydrodynamic size-distribution of AuNRs does not provide direct information on either the length or diameter of the AuNRs^49^. Nevertheless, the DLS measurements confirm that AuNRs are well-dispersed in solution, and show a consistent shift to a larger size as PEG is grafted on the AuNRs. We note that the first peak in Fig. 4(C) at less than ~5 nm has been associated with rotational diffusion of nonspherical NPs^50,51^, while the second and weaker peak is associated with translational diffusion coefficient of an effective spherical NP with a hydrodynamic diameter with size distribution that falls between the length and the diameter of the rods^49^. In our case, bare AuNR, PEG2K-AuNR, and PEG5K-AuNR have an effective diffusion coefficient of spherical AuNPs with 37 nm, 50 nm, and 58 nm hydrodynamic diameters, respectively (see discussion for Fig. S4). The DLS results confirm that grafting with PEG is successful as they clearly show that the effective hydrodynamic size of PEG-AuNRs increases with the molecular weight of PEG, as expected. The DLS also demonstrates that the grafted PEG-AuNRs are water-soluble and well dispersed in the suspension. We also note that UV-vis spectra show a tiny but measurable red-shift from 807 to 810 nm (for grafted AuNR, see Fig. S5) due to surface modification, in support of successful grafting by PEG.

PEG-functionalized AuNRs were further characterized by small angle X-ray scattering (SAXS) using 12ID-B beamline at the Advanced Photon Source (APS) at Argonne National Laboratory with instrumental setup similar to the one employed previously^52,53^. Figure 4(D) shows 1D SAXS data of AuNRs before (red triangles) and after functionalization with PEG (bare blue circles). We note that the SAXS profiles (i.e., intensity versus momentum transfer $Q$) for the bare AuNRs and PEG5K-AuNRs are dominated by the high electron density (ED) of the Au while, within uncertainties, the outer-shell PEG possesses ED that is practically the same as that of the aqueous medium, with no contrast between the PEG corona and the medium surrounding the particles^52^. Our best fit to the SAXS profiles, using a right circular cylindrical form factor^54^ assuming monodispersed rods, yields a diameter, $D=11.8\pm 0.4\,{\rm{nm}}$ and a length, $L=50.0\pm 9.0\,{\rm{nm}}$ (see Table 1) consistent with values provided by the manufacturer, and those obtained by the STEM images.

To determine the structure of the assemblies at the suspension/vapor interface we use surface sensitive synchrotron X-ray diffraction techniques^44^. Synchrotron X-ray measurements, including specular X-ray reflectivity (XRR) and grazing incidence small angle X-ray scattering (GISAXS) were conducted on beamline 15 ID-C, (at APS) using the liquid surface spectrometer (LSS) with incident X-ray beamenergy = 10 keV (wavelength, *λ* = 1.24 Å). We have adopted the experimental setup and analysis as those described elsewhere^55^. Briefly, the PEG-AuNRs suspension (about 1.5 mL) was introduced into a stainless steel trough of dimensions 20 × 100 × 0.3 mm^3^ in an enclosed chamber that is constantly flushed with water saturated helium at room temperature. To adjust the salt concentration of the PEG-AuNRs suspension, small amounts of highly concentrated salt solutions (NaCl) were applied sequentially to the trough for an incubation time of ~30 minutes before the XRR and GISAXS were collected. The GISAXS and XRR were collected with a PILATUS area detector (in two separate modes on the same spectrometer on the same samples sequentially in time). The XRR was collected over 25–35 min. where most of the time was used for motor movements and waiting-time for the surface to relax from the mechanical vibrations. The GISAXS was collected over 5 seconds per frame and two GISAXS shots were collected from different parts of the sample (surface translated with respect to the incident beam) to check for homogeneity, and reproducibility. To minimize potential radiation damage from the high the X-ray flux, the XRR and GISAXS did not start but after the enclosed box that includes the trough with the sample was purged with water saturated helium for a while, monitoring the O_2_ level coming out until it is at a level of 1–2% of the initial readout. Also for reproducibility, we collect data on different parts of the surface by translating the trough with respect to incident beam.

## Supplementary information

Supplementary Information.
