# Supplementary material for: Salt Mediated Self-Assembly of Poly(ethylene glycol)-Functionalized Gold Nanorods
Source: Sci Rep. 2019 Dec 30;9:20349. doi: 10.1038/s41598-019-56730-2 (PMC6937238; doi:10.1038/s41598-019-56730-2)
Supplement: Supplementary file 1 — Supplementary Information. [file 41598_2019_56730_MOESM1_ESM.pdf]

Supporting Information
Salt Mediated Self-Assembly of Poly(ethylene glycol)-Functionalized Gold Nanorods

Hyeong Jin Kim,* Wenjie Wang, Wei Bu, Md Mir Hossen, Alejandra Londono-Calderon, Andrew C Hillier, Tanya Prozorov, Surya Mallapragada, and David Vaknin*

E-mail: kimhj@iastate.edu; vaknin@ameslab.gov

XRR and GISAXS data for PEG5K-AuNRs with 2 M NaCl

The effect of salt concentration is further explored by increasing the concentration of NaCl up to 2 M. The first maximum in R/R_{F} data and extracted ρ_{2D} ED profile show small increase of PEG-AuNRs at the interface as the concentration of NaCl increases. In spite of slightly increased ED profile at the 2 M NaCl, the thickness of ED stratum is maintained, indicating that a uniform single PEG5K-AuNR film is formed. As with the XRR, the intensity of the GISAXS linecut profile is also slightly increased at the 2 M NaCl compared to the lower concnetrations.

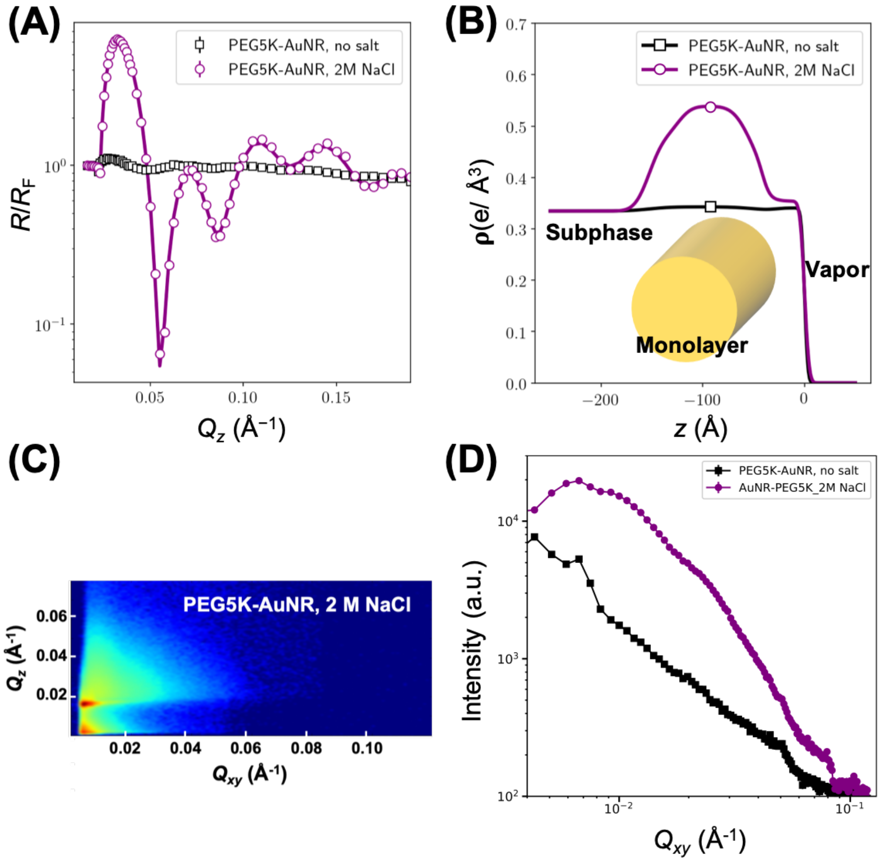

Figure S1: (A) Normalized XRR data and (C) 2D GISAXS patterns (Q_{xy},Q_z) for PEG5K-AuNRs with 2 M NaCl. Solid line of R/R_{F} in (A) is obtained from one of the best fit electron density (ED) profile shown in (B). (D) Horizontal Q_{xy} linecut profiles (integrated over a Q_z range from 0.02 to 0.1 Å^{-1}) from (C).

Estimation of surface coverage of assembled PEG-AuNRs

To determine the extent of accumulation of AuNRs at the interface, the excess electron surface density (ρ_{2D}) is calculated by using equation as follows,

$$\rho_{2D} = \int_{-\infty}^{0} (\rho_z - \rho_{water})\, dz.$$

ρ_{2D} of PEG5K-AuNRs and PEG2K-AuNRs are 14.4 and 56.3 e/Å^2, respectively. ρ_{2D} is higher for PEG2K-AuNRs than that of PEG5K-AuNRs, indicating aqueous surface is more populated with PEG2K-AuNRs, which is consistent with the smaller lattice constants (d_1, d_2) in PEG2K-AuNRs.

Next, we estimate the surface coverage of PEG-AuNRs with the maximum electron den-

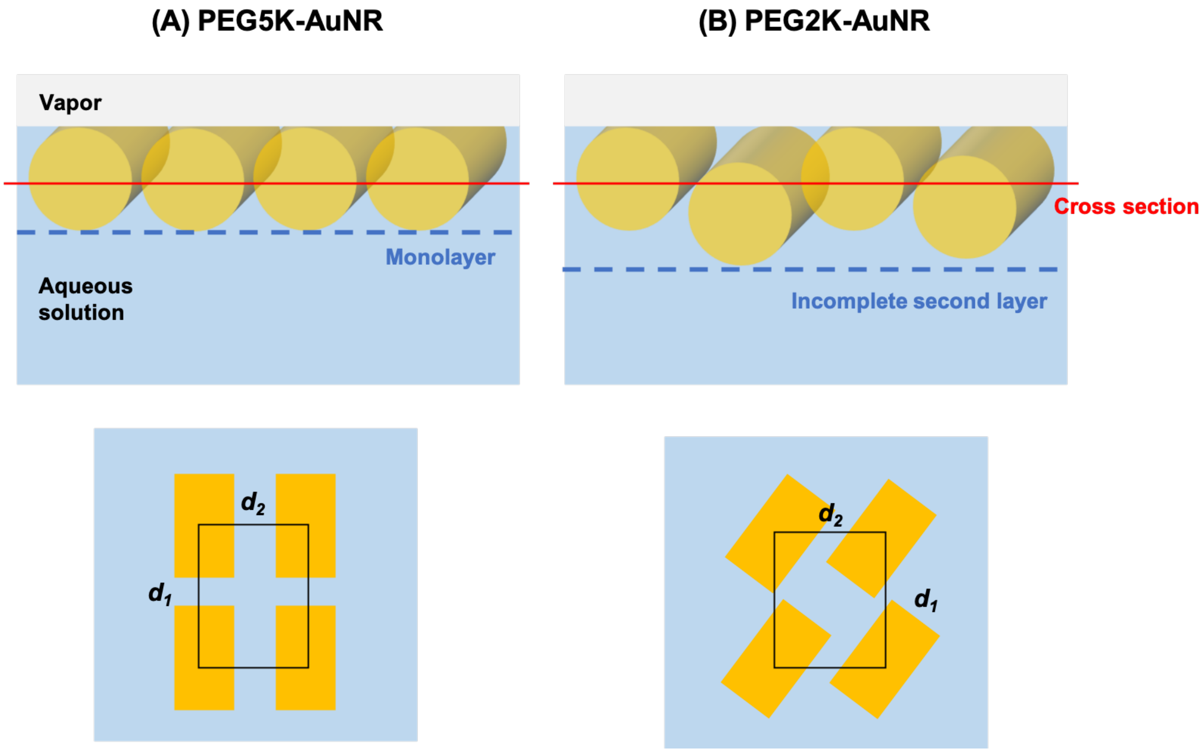

Figure S2: Schematic illustration of (A) PEG5K-AuNR and (B) PEG2K-AuNR layers at the vapor/liquid interface and the corresponding sectional views at the maximum electron density.

sity (ED) by using a space filling model. We assume that all AuNRs are ideally packed as shown in Figure S2, and the maximum ED position is calculated. For PEG5K-AuNRs, the maximum ED position is the center of AuNRs and its corresponding sectional view is illustrated in Figure S2(A). In this plane at the maximum ED of PEG5K-AuNRs, the area fraction of AuNRs in the 2D crystalline is calculated as follows,

The area fraction of AuNRs, $\phi = \mathrm{A}_{AuNR} \ / \ \mathrm{A}_{2D}$,where A_{AuNR} is the area occupied by an AuNRs and A_{2D} is the area of a 2D crystalline with lattice constants (a_1, a_2) from GISAXS.

$\mathrm{A}_{AuNR} =$ length $\times$ diameter of AuNRs $= 50 \times 11.8 = 590$ nm^2

$\mathrm{A}_{2D} = d_1 \times d_2 = 63.4 \times 25 = 1585$ nm^2

$\phi = \mathrm{A}_{AuNR} \ / \ \mathrm{A}_{2D} = 0.372$

With known electron densities of pure gold (ρ_{Au}) and subphase area of 50mM NaCl solution (ρ_{sub}), the maximum ED of 2D crystalline is estimated.

$\rho_{2Dmax} = \rho_{Au} * \phi + \rho_{sub} * (1 - \phi) = 1.94$ e/Å^3

Therefore, the surface coverage ($\psi = (\rho_{max} - \rho_{sub})/(\rho_{2Dmax} - \rho_{sub})$) of PEG5K-AuNRs can be determined by using both maximum ED of 2D crystalline (ρ_{2Dmax}, estimated from GISAXS lattice constants) and maximum ED (ρ_{max}, extracted from XRR) and it is 9.27 %.

Using same methods as that in PEG5K-AuNRs, the surface coverage of PEG2K-AuNRs is estimated as 18.4 % and the maximum surface coverage is approximately 40.0 %, assuming that all the nanorods in nanorod suspensions with a concentration of 1.5 nM have migrated to the vapor/liquid interface. As with the result from excess electron surface density (ρ_{2D}), the result from surface coverage estimation suggests that more PEG2K-AuNRs migrates and accumulates at the vapor/liquid interface than PEG5K-AuNRs. Since we have estimated the surface coverage of PEG-AuNRs by assuming an ideal 2Dassembly of PEG-AuNRs and using simple model, our estimated surface coverage values may differ from the actual values. However, we note that it would be an effective way to compare 2D assembled PEG5K-AuNRs and PEG2K-AuNRs easily.

STEM image of PEG5K-AuNRs

Figure S3 shows STEM image of dried drops of PEG5K-AuNRs without salt showing non-rod like particles.

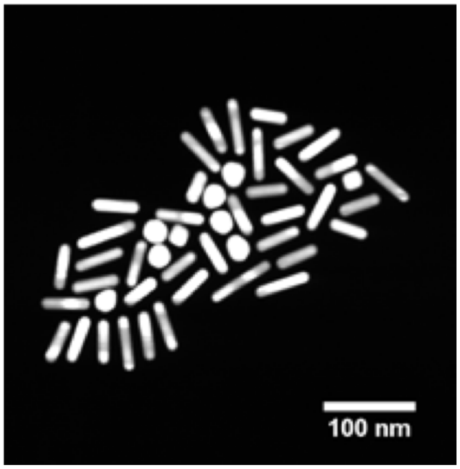

Figure S3: Representative STEM image of PEG5K-AuNRs drop-dried on TEM grid without salt.

Hydrodynamic diamter of bare AuNRs and PEG-AuNRs

Figure S4 shows the schematic illustration of hydrodynamic diameter of bare AuNRs, PEG2K-AuNRs, and PEG5K-AuNRs. The actual length of PEG polymer attached to the AuNR surface is much longer than the one depicted in Figure S4.

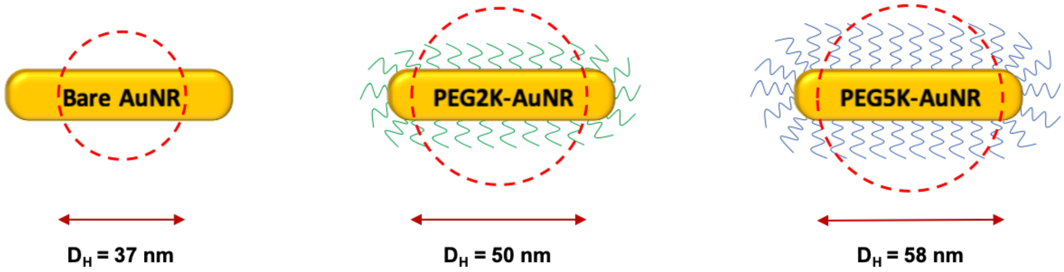

Figure S4: Schematic illustration of hydrodynamic diameter of bare AuNRs, PEG2K-AuNRs, and PEG5K-AuNRs.

UV-vis spectroscopy of PEG-AuNRs.

Absorption spectra of PEG-AuNRs were acquired using Thermo Fisher NanoDrop One. A 2 μl drop of PEG-AuNR solution was placed on NanoDrop pedestal to measure the UV-vis spectrum against deionized water as the reference. UV-vis spectra show a measurable red-shift from 807 to 810 nm (for bare and grafted AuNR, respectively; UV-vis spectra of bare AuNRs is provided by the manufacturer) due to surface modification, in supports of successful grafting by PEG.

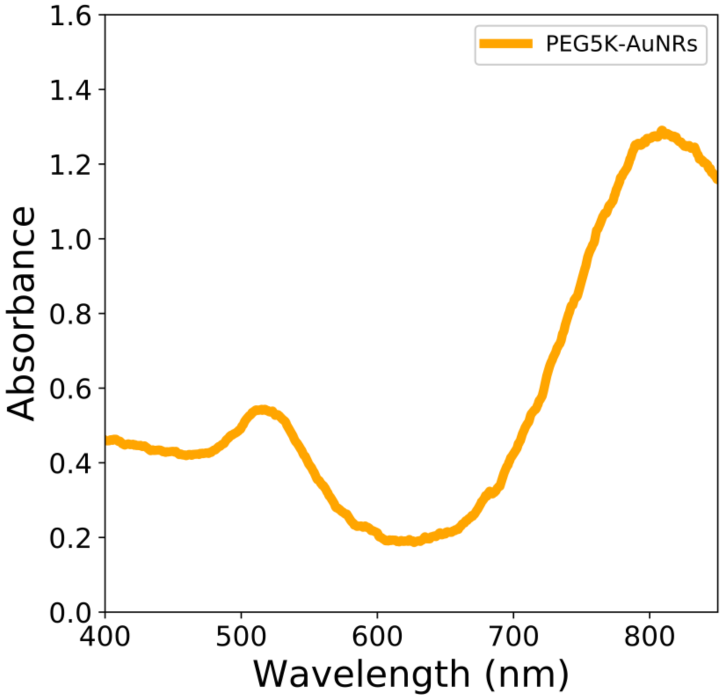

Figure S5: UV-VIS spectrum of PEG-AuNRs.
